# Has the time come for regional periprosthetic joint infection centers in the United States? A first-year experience

**DOI:** 10.5194/jbji-7-51-2022

**Published:** 2022-03-01

**Authors:** Murillo Adrados, Michael M. Valenzuela, Bryan D. Springer, Susan M. Odum, Thomas K. Fehring, Jesse E. Otero

**Affiliations:** 1 OrthoCarolina – Hip and Knee Center, Charlotte, NC 28209, USA; 2 OrthoCarolina Research Institute, Charlotte, NC 28209, USA; 3 Atrium Health Musculoskeletal Institute, Charlotte, NC 28204 USA

## Abstract

Several fields of orthopedics have concluded benefits from volume thresholds. We postulate that we could similarly optimize periprosthetic joint infection (PJI) treatment by creating a regional referral center, concentrating expertise and resources. Here, we review our reasoning and our first-year experience of a PJI referral center in the United States.

## 1 Viewpoint

Arthroplasty value and efficiency can be increased by the concentration of care in high-volume centers (Hollenbeck et al., 2020; Schwartz et al., 2020). While there is precedence of improved outcomes in complex orthopedic care at US specialty centers, in areas such as spine metastasis and sarcoma care, none exist for the treatment of periprosthetic joint infection (PJI) (Lazarides et al., 2019; Malik et al., 2021).

This is despite a similarly complex treatment algorithm, necessitating close, multidisciplinary collaboration, and a heavy, increasing burden of disease, projected to reach USD 1.85 billion by the year 2030 (Premkumar et al., 2021). As well established in sarcoma care, PJI surgical treatment initiated prior to referral to a tertiary center leads to worse 2-year infection-free survival (Tetreault et al., 2017). A PJI referral center could improve the success rate of PJI treatment by concentrating resources and expertise, and limiting the current care fragmentation.

This concept is not new in Europe. Recognizing the potential clinical and economic value of tertiary centers devoted to bone and joint infections, the French Health Ministry established a national network of 24 centers. In a review of their earliest work, Ferry at al. (2019) published on their first 5-year experience, from 2012 to 2017, where 19 961 bone and joint infections were treated, of which 7585 (38 %) were PJIs. They concluded that the establishment of the network promoted education and research, and it optimized the management of patients with bone and joint infections.

We postulated that a higher-volume center focused on the care of PJI through evidence-based approaches could similarly increase the value of treatment here in the United States. With this goal in mind, in 2019 we launched a PJI center and invited regional surgeons to refer their cases to our facility. We experienced a significant demand for care and quickly built a steady in-flow of referrals from the surrounding region.

From August 2019 through July 2020 we performed 298 PJI-related surgeries on 212 patients. Most patients had late chronic infections, 160 (75.5 %), with 34 (16.0 %) acute hematogenous infections and 18 (8.5 %) early post-operative infections. The average distance traveled for referred patients was 91.9 km (interquartile range 41.0–189.7​​​​​​​).

Most of the patients treated at the first-year PJI center had significant comorbidities. The average BMI was 31, and the vast majority of patients had serious compromising medical issues (74.5 %) and compromising local extremity factors (72.2 %).

This is reflective of the patient population at risk for PJI, but it also highlights the increased complexity and cost associated with treating such patients (Ong et al., 2009). The diversity of infections encountered also highlights the treatment complexity; 39 % of the infections treated were polymicrobial and 39 different fungal and bacterial species were identified throughout the year.

One of the challenges of the referral system is matching patient expectations with the reality of the PJI treatment process. We do not have a one-size-fits-all approach for managing infection, and if patients present with fixed expectations of immediate surgery, the shared decision making can be complicated and result in some patient dissatisfaction, especially patients traveling long distances. This dissatisfaction can start at the very initial visit, as patients presented with a presumed diagnosis of infection without an appropriate workup or outside notes. To begin to understand the magnitude of this issue, we determined the frequency with which a patient presents with a complete workup for PJI. Fewer than half of patients referred to our outpatient clinic (42.9 %) had what was deemed a minimum complete workup of erythrocyte sedimentation rate, C-reactive protein, and joint aspiration with cell count and culture. This is remarkably similar to the deficit that was reported by Tetreault et al. (2017). Education for referring physicians about their responsibilities prior to sending their patients remains a challenge and is a priority for our center.

Similar to the French experience, we expect to show the same increase in value by concentrating the care of PJI. We expect this will also significantly improve the economics of PJI treatment. Higher efficiency and improved outcomes may decrease the high economic toll of PJI treatment (Kurtz et al., 2012). Ideally, a national network of PJI centers, if established, has the potential for significant economic benefit to the health care system as a whole. Such centers would have economies of scale and a more experienced interdisciplinary team, which should lead to fewer operative interventions and lower costs.

An additional benefit of having a PJI referral center is the concentration of clinical data for research. We have multiple prospective trials specifically focused on the treatment of PJI. The referral system allows for strong enrollment into our randomized clinical trials and our PJI registry. Some of our ongoing prospective projects include the following:

- randomized one stage versus two stage for periprosthetic hip and knee infection,
- whether intraosseous antibiotics improve the results of irrigation and debridement and prosthetic retention for PJI,
- a randomized controlled trial of alternating irrigation of vancomycin and tobramycin sulfate in patients undergoing two-stage exchange arthroplasty for periprosthetic joint infection of the hip and knee, and
- whether antibiotic loaded intramedullary dowels are necessary in two-stage resection knee arthroplasty.

In addition, we have numerous retrospective studies arising from our PJI-specific database, comprising more than 3400 periprosthetic infection surgeries.

## 2 Conclusion

We have shown that the establishment of a PJI center is feasible and well accepted by patients who are willing to travel for specialized care. In fact, in the second year of the PJI center, from August 2020 to July 2021, 279 known PJI surgeries took place. It is also well accepted by referring surgeons who may only encounter a PJI a few times a year. A PJI center provides an outlet for such surgeons unfamiliar with the nuances of PJI treatment, allowing referral to a center where multiple infection-related surgeries are performed weekly. It is our hope that our center will provide a model for other regional PJI centers that use evidence-based treatment protocols, have experienced multidisciplinary treatment teams, and perform multicenter-related PJI research. We believe the time has come for regional periprosthetic joint infection centers in the United States.

## Data Availability

Data are available on request from the authors.

## References

[bib1.bib1] Ferry T, Seng P, Mainard D, Jenny JY, Laurent F, Senneville E, Grare M, Jolivet-Gougeon A, Bernard L, Marmor S (2019). CRIOAc network. The CRIOAc healthcare network in France: A nationwide Health Ministry program to improve the management of bone and joint infection. Orthop Traumatol Surg Res.

[bib1.bib2] Hollenbeck B, Hoffman MA, Tromanhauser SG (2020). High-Volume Arthroplasty Centers Demonstrate Higher Composite Quality Scores and Enhanced Value: Perspective on Higher-Volume Hospitals Performing Arthroplasty from 2001 to 2011. J Bone Joint Surg Am.

[bib1.bib3] Kurtz SM, Lau E, Watson H, Schmier JK, Parvizi J (2012). Economic burden of periprosthetic joint infection in the United States. J Arthroplasty.

[bib1.bib4] Lazarides AL, Kerr DL, Nussbaum DP, Kreulen RT, Somarelli JA, Blazer III DG, Brigman BE, Eward WC (2019). Soft Tissue Sarcoma of the Extremities: What Is the Value of Treating at High-volume Centers?. Clin Orthop Relat Res.

[bib1.bib5] Malik AT, Khan SN, Voskuil RT, Alexander JH, Drain JP, Scharschmidt TJ (2021). What Is the Value of Undergoing Surgery for Spinal Metastases at Dedicated Cancer Centers?. Clin Orthop Relat Res.

[bib1.bib6] Ong KL, Kurtz SM, Lau E, Bozic KJ, Berry DJ, Parvizi J (2009). Prosthetic joint infection risk after total hip arthroplasty in the Medicare population. J Arthroplasty.

[bib1.bib7] Premkumar A, Kolin DA, Farley KX, Wilson JM, McLawhorn AS, Cross MB, Sculco PK (2021). Projected Economic Burden of Periprosthetic Joint Infection of the Hip and Knee in the United States. J Arthroplasty.

[bib1.bib8] Schwartz AM, Farley KX, Nazzal EM, Manz WJ, Bradbury TL, Guild GN (2020). Evidence-Based Hospital Procedural Volumes as Predictors of Outcomes After Revision Hip Arthroplasty. J Arthroplasty.

[bib1.bib9] Tetreault MW, Estrera KA, Kayupov E, Brander C, Della Valle CJ (2017). Are patients being evaluated for periprosthetic joint infection prior to referral to a tertiary care center?. Arthroplast Today.

